# When to Start Dialysis in Elderly Patients

**DOI:** 10.5812/numonthly.14288

**Published:** 2013-08-25

**Authors:** Zohreh Rostami

**Affiliations:** 1 Nephrology and Urology Research Center, Baqiyatallah University of Medical Sciences, Tehran, IR Iran

**Keywords:** Quality of Life, Renal Dialysis, Aged

End stage renal disease (ESRD) is a potentially treatable chronic disease that depends on the dialysis machine long life. Although patient survival is an important factor, it is not enough for these cases and the physicians also try to improve quality of life in this population. Certainly, quality of life is determined by so many conveniences influence on mental, social and spiritual well-being. Dialyzed patients have a great deal of limitations in activities, job, foods and have dependencies to physicians, dialysis staff, dialysis machines, family members which influence on the different aspects of their quality of life and life satisfaction (1). These problems become more prominent when taking to account elderly patients with advanced chronic kidney disease (CKD) which form the fastest growing part of the ESRD patients. Although age itself is not a contraindication for kidney transplantation and old age recipients has increased over time (2), in these individuals comorbidities are common which prone them to post transplantation complications. Subsequently, the majority of elderly patients are ineligible for transplantation therefore stays on dialysis for the remainder of their lives (2).

This expanding part of ‘‘geriatric’’ dialysis patients have special kinds of problems that need specific information. Kidney disease is only one of the many conditions affecting their lives (3, 4). Moreover, they do not always good filling on renal replacement therapy (5). To date, there is a challenge for the nephrologist to decide when dialysis initiation can both prolonged life and improve quality of life especially while there are multiple comorbidities in elderly patients. The optimal timing of initiation of dialysis remains an unresolved issue among nephrologists (6). When is it the best time to start dialysis in elderly patients? Early or late starting? Really, there are some conflicting data for decision making.

During the last decade, several registries report a historical tendency to early initiation of dialysis which is associated with decreased mortality (6-9). However, the other observational studies have been unable to conﬁrm any beneﬁt of early start dialysis (6, 10). In contrast, these studies showed that patients who beginning the dialysis at a lower estimated glomerular ﬁltration rate (eGFR) lived signiﬁcantly longer (6, 11), and the patients starting early had an average of 6 months longer on dialysis (2). This contrasts with the fact that comorbid conditions associated with late referral for treatment are poor prognostic factors in this population (5, 6, 12, 13). By the way, these patients often come late for dialysis (2).

Recently, Cooper et al. in a randomized control study shows no significant difference between both early and late groups in the mortality rate and the frequency of adverse events, such as cardiovascular events, infections or complications of dialysis (14).

Since 2006 modified guidelines recommended that dialysis should be initiated before GFR < 15 mL/min if patient presents with symptoms suspected to be connected to a combination of current comorbidities and impaired renal function (6). In addition, dialysis should be noted in presence of following clinical conditions: uremic syndrome, poor control volume overload or hypertension, and progressive signs of protein–energy wasting (6). So, patients with symptoms or co-morbidity are more likely to be started on dialysis early (11).

In this editorial, we focused on bias and weakness of studies that adhere with late initiation of dialysis. One of the most important biases in these studies is a decision to start of dialysis based on eGFR from serum creatinine, by MDRD equation, Cockcroft and Gault’s equation or reciprocal creatinine plots (6) which none of them should not be used when the GFR is < 30 mL/min/1.73 m^2^ to determine the need for dialysis (11, 15).

On the other hand, serum creatinine concentration not only depends on residual renal function, but also depends on nutritional status, muscle mass and volume overload, which all relate inversely to residual renal function (9). Low serum creatinine level is also seen due to low muscle mass owing to inactivity, malnutrition and dilution in presence of volume overload. In all conditions patients will have higher co-morbidity, yet have a lower serum creatinine. So, eGFR will be overestimated and they are more probable to be included in ‘earlier’ start groups (11). However, renal function based on serum creatinine (as with eGFR) is useless or even misleading as a guide on when to start dialysis (11).

Survivor bias: this kind of bias causes the results of studies deviate toward higher because only patients who were strong enough to survive until the end of the period are included (16). 

In these studies, the CKD patients were only on acutely initiation of dialysis were included, while, so many of them died before dialysis to be initiated, possibly due to uremia, were excluded. Only the fittest patients living enough until they are included in the late start groups (11). On the other hand, these studies are prone to the ‘lead time bias’. Lead time defines as the length of time between the detection of a disease and its usual clinical presentation (17). Lead time bias occurs when the life span obtained by dialysis latency is not considered. This contributing factor will skew the results in favor of early start dialysis apparently (11, 18).

Nevertheless, patients who were included in the late start dialysis group, need a conservative care in lead time (18). Such ‘conservative’ care requires strict attention to the complications of uremia (i.e. abnormalities of nutrition, acid–base, fluid, bone and mineral metabolism and anemia). Furthermore, patients have large variations in their conservative care depending on country, region and attending nephrologist. As patients are more expected to be offered and receive conservative care in the UK than in the USA (2).

Elderly patients and patients with symptoms or co-morbidity are more likely to be presented repeatedly with absolute urgent dialysis indications rather than waiting for eGFR level less than a specific level. In this condition, really, decline dialysis could mean dying sooner and may not having enough time for dialysis preparation.

In Hwang et al. study (2010), patients who died during the first 90 days, as acute kidney injury, after beginning dialysis were excluded (19). Subsequently, they may excluded the patients with CKD who presented with acute symptoms and died just after starting dialysis in an emergency or a little time once maintained dialysis, because the start of dialysis was too late; as a result, some of the late beginners with the worst outcomes have not been enrolled in the analysis (9).

Although all studies adjusted for ‘comorbidity at the start of dialysis’, a part of the comorbidity adjustment for dialysis initiation might have been omitted if dialysis had started earlier (9).

Furthermore, definition used for comorbidity was different. Consequently, a young patient with fluid overload because of late dialysis start will receive the same label, ‘congestive heart failure’, as the elderly diabetic with CKD stage 4 who develops pulmonary edema, necessitating an emergency start of dialysis at an eGFR of 15 mL/min. It is clear that the prognosis differs widely among both of them, independent of eGFR at the initiation of dialysis. This example also illustrates that the term ‘early’ start is a misleading term if it is defined by eGFR, rather than by patient condition (9).

Consequently, we should either agree with the lag of start dialysis until anuric phase or accept that there is something wrong with the data and conclusions of these studies. If we accept the former, CKD stage 4-5 patients may die from uremia before they become anuric unless dialysis is done. However, in real life, decisions to start dialysis are, to a large extent, based on clinical parameters, so that patients underwent dialysis only when they become symptomatic. If the eGFR versus other elements used in mentioned studies to define early and late start for decision making, the accuracy of the definition of ‘early’ and ‘late’ can be questioned, and the conclusions would be meaningless too. There is definitely a need for a survey among physicians on the criteria they really use for starting dialysis (9). Future randomized control trials may help us to determine the optimal time to start dialysis.

Recommendations: If physicians use eGFR as a parameter to start dialysis, why is there such a different amount of eGFR for dialysis initiation in these retrospective cohort studies? Probably, physicians don’t use eGFR as a starting criterion (9). It’s a matter of debate. Then, what is that? 

Based on current knowledge the best time for initiation of dialysis is potentially depended on subjective and objective factors that may play an important role in determining patient outcomes and quality of life. In this view of point, elderly patients who become more symptomatic due to other comorbid disorders more likely to be switched on dialysis earlier than others (9). Decision making for dialysis schedule in this growing population is very difficult. Since being on dialysis may only prolong the period of their lives rather than providing any improvement in quality of living. 

In general, we recommend symptom based dialysis initiation policy should be considered. We ought to follow CKD patients for renal function deterioration and using well-timed dialysis to preserve other organs function such as heart and brain rather than waiting for complete renal shutdown prior to renal replacement (20), while the patients suffer from pericardial effusion, dementia, weight loss and so on. Such patients have exactly to be followed in renal clinics with conservative management focusing on anemia, fluid status and symptom control. Although according to the previous studies which are in disfavor of an early start (6, 21, 22), current clinical practice, as recommended by guidelines, is, in CKD patients with higher comorbidity start dialysis at higher eGFR. In fact, higher mortality is due to higher comorbidity, not the higher eGFR (18, 19). Therefore, considering the mentioned biases and weaknesses of studies it seems early dialysis start may be beneﬁcial, because in elderly patients it can inhibit poor outcome risk factors such as fluid over load, anemia, uremia, acidemia, electrolyte imbalances, and malnutrition and so on.

As so many nephrologists are ignoring eGFR in planning the start dialysis, it oﬀers an additional approach to identifying a starting point for dialysis such as risk stratiﬁcation based on comorbidity scores for death to low, medium, and high (5, 23).

In hemodialysis patients, poorer social support and other psychosocial factors leading to higher mortality risk, lower compliance to medical managements, higher rate of missed or shortened dialysis sessions and poorer physical function and quality of life (24). Furthermore, in geriatrics comorbidities, functional and cognitive decline increasing dependency to social and family support (1). Social support can be received from family members, friends, colleagues, and medical personnel (24). Then a close communication with the patient improves patient’s adherence to treatment (24).

The default dialysis modality for elderly patients with ESRD is hemodialysis which resulting to hemodynamic instability and poorly tolerated by elderly patients. All of these factors impact on the management of ESRD (2). Home daily or nocturnal dialysis could be better option (25, 26).
